# Prevalence and determinants of SHS exposure in public and private areas after the 2010 smoke-free legislation in Greece

**DOI:** 10.1186/1617-9625-12-S1-A16

**Published:** 2014-06-06

**Authors:** Sotiria Schoretsaniti, Filippos T Filippidis, Constantine I Vardavas, Chara Tzavara, Christine Dimitrakaki, Panagiotis Behrakis, Gregory N Connolly, Yannis Tountas

**Affiliations:** 1 Center for Health Services Research, Department of Hygiene, Epidemiology and Medical Statistics,School of Medicine, National and Kapodistrian University of Athens, Athens, 11527, Greece; 2 Center for Global Tobacco Control, Department of Social and Behavioral Sciences, Harvard School of Public Health, West Boston, 02115, Massachusetts, USA; 3 Smoking and Lung Cancer Research Center, Hellenic Cancer Society, Athens, 11521, Greece; 4 Foundation for Biomedical Research of the Academy of Athens, Athens, 115 27, Greece; 5 Department of Environmental Health, Harvard School of Public Health, 677 Huntington Avenue Landmark, West Boston, MA 02115, USA

## Background

The objective of the present survey was to assess the extent and socioeconomic determinants of population exposure to secondhand smoke (SHS) in Greece in 2011.

## Materials and methods

The national household survey Hellas Health IV was conducted in October 2011. SHS exposure was based on self-reported exposure within home, workplace and public places.

## Results

33.1% of the respondents reported living in a smoke-free home. Smokers (p<0.001) and single individuals (p<0.017) were less likely to prohibit smoking at home. SHS exposure at work, in restaurants and in bars/clubs/cafes was frequently mentioned by 41.6%, 84.2% and 90.5% respectively. SHS exposure in a bar/club/café was noted more among single individuals (p=0.004) and those aged 18-34 years (p=0.007).

## Conclusions

Inhabitants of rural areas were more likely to report someone smoking indoors in all the above venues. Public health education and effective enforcement of the nationwide smoke-free legislation are imperative.

